# Ergothioneine rescues obesity-induced testicular dysfunction via dual restoration of steroidogenesis and mitochondrial redox homeostasis

**DOI:** 10.1016/j.redox.2026.104090

**Published:** 2026-02-18

**Authors:** Xiaomin Li, Jiajing Lin, Man Wu, Feixue Han, Shuyan Chen, Hongfei Ke, Zhiying Huang, Tianwen Peng, Yu Lan, Xin Fu, You Che, Zhicong Chen, Geng An

**Affiliations:** a Department of Obstetrics and Gynecology, Center for Reproductive Medicine, Guangdong Provincial Key Laboratory of Major Obstetric Diseases, Guangdong Provincial clinical Research Center for Obstetrics and Gynecology, Guangdong Hong Kong-Macao Greater Bay Area Higher Education Joint Laboratory of Maternal-Fetal Medicine, The Third Affiliated Hospital, Guangzhou Medical University, Guangzhou, China; b HKU-Pasteur Research Pole, School of Public Health, Li Ka Shing Faculty of Medicine, The University of Hong Kong, HongKong, China; c The First People's Hospital of Zhaoqing, Zhaoqing, Guangdong, China

**Keywords:** Ergothioneine, Obesity-induced testicular dysfunction, Oxidative stress, Steroidogenesis, Mitochondrial redox balance

## Abstract

**Background:**

Although obesity is closely linked to reduced male fertility, the specific testicular metabolic and redox mechanisms driving impaired spermatogenesis remain elusive.

**Methods:**

Using a high-fat diet (HFD) mouse model, combined with multi-omics profiling, cellular assays, and ex vivo human testis cultures, we show that chronic HFD feeding progressively disrupts sperm quality, seminiferous architecture, and steroidogenic capacity.

**Results:**

Despite unchanged testis weight, HFD significantly reduced sperm density by 21.6% and motility by 44.9%. Transcriptomic and metabolomic analyses revealed a marked suppression of oxidative phosphorylation and depletion of steroidogenic intermediates. Notably, ergothioneine (ET) was identified as the only metabolite consistently 8 reduced across time-course analyses, highlighting its potential as a testis-intrinsic biomarker of cumulative redox stress. ET supplementation (100 mg/kg/day) markedly restored seminiferous epithelial organization and increased the expression of spermatogenic markers. Functionally, ET alleviated the intracellular oxidative burden by reducing lipid peroxidation (TBARS levels decreased by 1.5-fold), and restoring antioxidant enzyme activities. ET enhanced mitochondrial stability, preserving mitochondrial membrane potential (ΔΨm) and reducing mitochondrial superoxide (O_2_^• -^) overproduction. Mechanistically, ET reactivated the canonical PKA-CREB-StAR signaling cascade in Leydig cells, reinstating androgen biosynthesis (in vivo DHT increased 1.3-fold, *P* < 0.01). Finally, ex vivo human testis cultures confirmed that ET attenuated oxidative stress indicators (reducing fluorescence intensity by 2.1-fold) and enhanced testosterone release by 1.4-fold.

**Conclusion:**

These findings establish progressive ET depletion as a hallmark of obesity-induced testicular dysfunction and demonstrate that ET supplementation restores steroidogenesis and mitochondrial redox homeostasis, providing a robust mechanistic basis for antioxidant-guided interventions in male infertility.

## Highlights

• Chronic HFD triggers intrinsic redox-metabolic reprogramming in the testis, disrupting steroidogenic homeostasis.
• Integrate metabolomics and RNA-seq to reveal mitochondrial dysfunction and oxidative stress in testes.
• Identify a progressive decline of l-ergothioneine with HFD, nominating a diet-derived redox biomarker.
• ET rescues steroidogenesis via reactivating the PKA-CREB-StAR pathway in Leydig cells.

## Graphical abstract

Preventive ET supplementation restores StAR-mediated steroid hormone transport, stabilizes mitochondrial/redox homeostasis, and rescues steroidogenic function.

**Figure:**
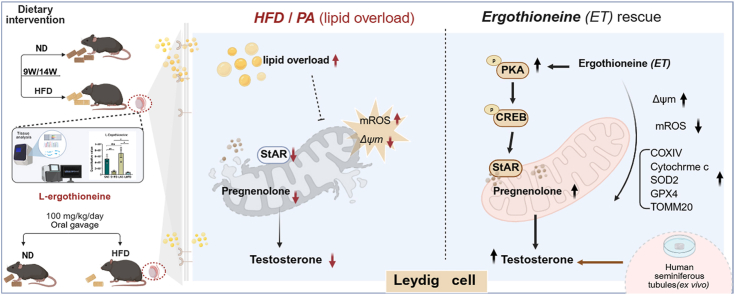

## 1 Introduction

Obesity is a major public health problem and a well-established risk factor for male infertility, with numerous epidemiological and clinical studies reporting reduced semen quality, altered hormone profiles, and increased risk of reproductive failure in obese men [[1], [2], [3]]. The development of obesity involves genetic, environmental, and lifestyle factors, among which a high-calorie diet a major driver of metabolic disorders in humans and animals [[4], [5], [6], [7]]. Excess adipose tissue elevates aromatase activity [8], enhancing the conversion of androgens to estrogens [9], thereby generating a hormonal milieu of high estrogens and low androgens that suppresses testosterone secretion and impairs spermatogenesis [1,[10], [11], [12]]. In parallel, obesity is characterized by leptin and insulin resistance, systemic low-grade inflammation, and a persistent state of oxidative stress, defined by excessive intracellular oxidative burden generation and diminished antioxidant defenses [13,14]. These alterations disrupt the testicular microenvironment by inducing lipid peroxidation, mitochondrial injury, and DNA damage, which collectively compromise sperm motility, fertilization capacity, and genomic integrity [[15], [16], [17], [18]]. Despite the recognition of these systemic factors, the intrinsic testicular metabolic and redox mechanisms underlying obesity-associated infertility remain incompletely understood.

Traditional interventions for obesity, including lifestyle modifications and pharmacological therapies, can partially restore metabolic homeostasis and improve sperm function [[19], [20], [21]]. However, long-term adherence to restrictive diets and exercise regimens is challenging, while pharmacological options such as orlistat, liraglutide, and lorcaserin often cause adverse effects [[22], [23], [24], [25], [26]]. Bariatric surgery is effective but invasive and reserved for severe obesity [27,28]. These limitations underscore the need for novel, safe, and effective therapeutic strategies, among which natural dietary compounds have attracted increasing attention. In particular, dietary antioxidants are emerging as promising candidates, given their capacity to counteract oxidative stress and inflammation.

Recent advances in omics technologies, particularly metabolomics and transcriptomics, provide powerful tools to map biochemical and transcriptional changes in disease states [[29], [30], [31]]. In male infertility research, metabolomics has revealed novel biomarkers and therapeutic targets [32,33], yet most studies focus on seminal plasma rather than direct testicular alterations. Consequently, the metabolic characteristics of obesity-induced testicular dysfunction remain poorly defined, and testis-intrinsic regulatory networks linking metabolic stress to defective spermatogenesis are largely unexplored.

Here, we employed a classical HFD-induced mouse model to systematically map the testicular metabolic landscape under obesity. By integrating untargeted metabolomics and RNA-seq, we identified progressive metabolic and transcriptional remodeling that disrupts steroidogenesis, mitochondrial function, and redox balance. Among the altered metabolites, ergothioneine (ET) emerged as the only compound that declined progressively with HFD exposure. ET is a naturally occurring thiol amino acid enriched in mushrooms and other dietary sources, and it acts as a potent antioxidant that accumulates in metabolically active and oxidative stress–prone tissues [34,35]. Building on this observation, we further evaluated ET supplementation as a redox-active intervention in vivo, in vitro, and in ex vivo human testis models. This integrative framework not only provides mechanistic insights into obesity-related reproductive decline but also highlights ET as both a biomarker candidate and a therapeutic adjunct for obesity-associated male infertility.

## 2 Materials and methods

### 2.1 Animals and experimental design

Animals were housed under controlled conditions (25 ± 2 °C; 55 ± 10% relative humidity) with a 12 h light/dark cycle and ad libitum access to food and water. Mice colonies were maintained in a specific pathogen-free animal facility. The mice used in the study were sourced from two different suppliers, corresponding to two distinct animal models: i) The high-fat diet (HFD)-induced obesity model mice were obtained from Shanghai Model Organisms Center, Inc. (Shanghai, China). These mice were further divided into a normal diet control group (Control) and a high-fat diet group (HFD). Based on the duration of HFD feeding, the HFD group was subdivided into a short-term HFD subgroup (9 weeks) and a long-term HFD subgroup (14 weeks). ii) Seven-week-old male C57BL/6J mice were purchased from Seyotin Animal Co., Ltd. (Guangzhou, China). Animal care and procedures complied with relevant regulations and were approved by the Institutional Animal Care and Use Committee of Guangzhou Seyotin Biotechnology Co., Ltd. (approval no. SYT2024090). After a one-week acclimation, male mice were randomly assigned to three groups (n = 6 per group): mice standard chow (Normal Diet, Control), high-fat diet (HFD), and high-fat diet plus Ergothioneine (HFD + ET). Mice in the HFD group received diet D12492 (Research Diets Inc., New Brunswick, NJ, USA; 60% kcal from fat). The HFD + ET group was fed the same diet and, in parallel, administered Ergothioneine (497-30-3; Med Chem Express) by oral gavage at 100 mg/kg/day. The ET intervention was implemented concurrently with HFD feeding for 8 weeks.

### 2.2 Sperm parameters measurement in mice

Sperm parameters were assessed as previously described [36]. Epididymides were dissected from each mouse and transferred to HTF medium. Surrounding adipose tissue was carefully removed, and the epididymides were minced into small pieces using micro scissors. After a 5-min incubation at 37 °C, the tissue fragments were further shredded in preheated G-IVF PLUS medium (10136; Vitro life, Sweden) at 37 °C to release sperm into the medium. The resulting sperm suspension was incubated for an additional 15 min at 37 °C to ensure uniform diffusion. Sperm concentration and motility were subsequently analyzed using a computer-assisted sperm analysis (CASA) system.

### 2.3 Histological analyses

Following animal euthanasia, testes were fixed in Bouin's fixative, dehydrated through a graded ethanol series, cleared in xylene, and embedded in paraffin. Tissue sections (8 μm thick) were stained with hematoxylin and eosin (H&E) for evaluation under light microscopy. Micrographs were captured using an upright fluorescence microscope (Leica DM 3000). The thickness of the germinal epithelium (GE) and the mean testicular biopsy score (MTBS) were calculated using Image J software. Testicular spermatogenesis was determined according to Johnsen's score [37]. For each animal, at least 100 cross-sectioned seminiferous tubules were randomly selected and and scored on a scale of 1 to 10 based on the histological criteria detailed in Table 2. To ensure the objectivity of the evaluation, scoring was performed by two independent pathologists in a blinded manner, without knowledge of the experimental groups. The MTBS was calculated as the average score of all evaluated tubules per testis. Additionally, the GE thickness was measured from the basement membrane to the lumen in 20 round-sectioned tubules per mouse using ImageJ software.

### 2.4 Immunohistochemical staining

Paraffin-embedded mouse testis sections (8 μm) were baked, deparaffinized, and rehydrated. Heat-induced epitope retrieval was performed in Tris-EDTA buffer using an autoclave/pressure cooker at 121 °C under full pressure for 3 min, followed by natural depressurization and cooling to room temperature for 30 min. Endogenous peroxidase was quenched with 3% H_2_O_2_ (10 min), and non-specific binding was blocked with normal serum (30 min, RT). Slides were incubated overnight at 4 °C with rabbit primary antibodies to TNP1 (condensing spermatids), γH2AX (meiotic spermatocytes) and HSD17B11 (steroidogenic enzyme) at vendor-recommended dilutions (details in Supplementary Table 1). After TBS-T washes, an HRP-conjugated goat anti-rabbit secondary antibody was applied for 1 h at RT. Signal was developed with DAB, counterstained with hematoxylin, dehydrated, cleared, and cover slipped. Quantitative analysis of immunohistochemical staining was performed using ImageJ software.

### 2.5 Bodipy staining

Adherent cells were fixed by immersion in ice-cold 4 % paraformaldehyde for 10 min. Then, the confocal dishes were washed three times in deionized water and incubated for 30 min with the lipophilic dye Bodipy 493/503 (C2053S; Beyotime Biotechnology, Shanghai, China) at 37 °C. Digital photographs were captured from each section under fluorescence illumination, and the area content of the lipid droplet was quantified using ImageJ software.

### 2.6 Cell treatment

The mouse TM3 Leydig cell line (CL-0234; RRID: CVCL_4326), isolated from a male mouse, was obtained from Wuhan Procell in 2023. The cells were authenticated by short tandem repeat (STR) profiling and confirmed to be free of mycoplasma contamination before use in the experiments. The cells were cultured in DMEM/F12 medium supplemented with 5% horse serum, 2.5% fetal bovine serum, and 1% penicillin-streptomycin solution, under 5% CO_2_ at 37 °C with saturated humidity. To determine the optimal treatment concentrations of PA and ET, a concentration gradient of PA (Xi'an Kunchuang Technology Development Co., Ltd.) was applied to the cells, and cell viability was assessed after 48 h of co-culture. ET was directly diluted in the culture medium. TM3 stromal cells were pre-treated 24 h with ET (0.2 and 0.8 mM) and/or exposed to PA (20 μM) for 48 h for subsequent experiments.

### 2.7 Cell proliferation assay

The cell viability assay used a Cell Counting Method-8 kit (CCK8, R11011, TransGen Biotech). A total of 4 × 10^3^ cells/well were plated in 96-well plates, and the plates were at 37 °C, 5% CO_2_ incubator. After 48h of proliferation, CCK-8 solution was added and incubated at 37 °C for 1 h. The optical density (OD) value was measured at 450 nm using a microplate reader. Experiments were performed in five replicates. Cell viability was calculated according to the instruction manual formula.

### 2.8 RNA-seq and analysis

RNA integrity was assessed using the RNA Nano 6000 Assay Kit on the Bioanalyzer 2100 system (Agilent Technologies, CA, USA). Total RNA was used as the input material for RNA sample preparation. Briefly, mRNA was purified from total RNA using poly-T oligo-attached magnetic beads. Fragmentation was performed using divalent cations under elevated temperature in First Strand Synthesis Reaction Buffer (5X). First-strand cDNA was synthesized using a random hexamer primer and M-Mu LV Reverse Transcriptase (RNase H-). Second-strand cDNA synthesis was subsequently performed using DNA Polymerase I and RNase H. Remaining overhangs were converted into blunt ends through exonuclease/polymerase activity. Following adenylation of the 3′ ends of DNA fragments, adaptors with hairpin loop structures were ligated to prepare for hybridization. To select cDNA fragments preferentially 370-420 bp in length, the library fragments were purified using the AMPure XP system. PCR amplification was then performed with Phusion High-Fidelity DNA polymerase, universal PCR primers, and an Index (X) primer. Finally, PCR products were purified using the AMPure XP system, and library quality was assessed using the Agilent Bioanalyzer 2100 system. RNA-seq was conducted by Beijing Novogene Co., Ltd. Data analysis was performed using the Novogene Cloud platform(https://magic.novogene.com/). Protein-protein interaction assessment was conducted using the STRING database (https://string-db.org) [38] and modeled using Cytoscape [39].

### 2.9 Testosterone elisa

Testosterone levels in both conditioned media from TM3 Leydig cells and testicular tissue homogenates were quantified using a competitive ELISA kit (FineTest, Wuhan, China; Cat No. EU0400-HS). For TM3 cell supernatants, media were collected after treatments, clarified by centrifugation (1000×*g*, 10 min, 4 °C), aliquoted, and stored at −80 °C until analysis. Testicular tissues were homogenized in cold PBS, centrifuged (12,000×*g*, 20 min, 4 °C), and supernatants were collected and stored similarly. The assay was performed per manufacturer's instructions: standards or samples (50 μL, in duplicate) and HRP conjugate (50 μL) were added to antibody-coated wells, incubated for 60 min at 37 °C, washed five times, developed with TMB substrate (100 μL, 15 min, 37 °C in darkness), and stopped with stop solution (50 μL). Absorbance was measured at 450 nm with a reference wavelength of 630 nm. An 8-point standard curve (0–2000 pg/mL) was established using 4-parameter logistic (4 PL) regression for concentration interpolation. All sample concentrations were corrected for dilution factor. Intra-assay coefficients of variation were <10%. Samples exceeding the working range were re-assayed at appropriate dilutions.

### 2.10 Oil Red O staining

Neutral lipids in testicular tissue were detected using Oil Red O (ORO) staining on cryosections. Fresh tissues were embedded in O.C.T. compound, and 8-10 μm sections were prepared using a cryostat. Sections were air-dried, fixed in 10% formalin for 10 min, and rinsed with 60% isopropanol. They were then incubated in filtered 0.3% ORO solution (in 60% isopropanol) for 15 min at room temperature. After differentiation in 60% isopropanol and washing with water, nuclei were counterstained with hematoxylin. Sections were mounted with aqueous mounting medium and imaged under uniform bright-field microscopy conditions. Lipid droplet area was quantified using ImageJ software (NIH).

### 2.11 Analysis of metabolites using non-targeted metabolomics

Tissue samples (100 mg) were individually ground with liquid nitrogen, and the homogenate was resuspended in prechilled 80% methanol and vortexed thoroughly. The samples were incubated on ice for 5 min and then centrifuged at 15,000×*g* at 4 °C for 20 min. A portion of the supernatant was diluted to a final concentration containing 53% methanol using LC-MS-grade water. The diluted samples were transferred to fresh Eppendorf tubes and centrifuged again at 15,000×*g* at 4 °C for 20 min. Finally, the supernatant was injected into the LC-MS/MS system for analysis [40]. UHPLC-MS/MS analyses were performed using a Vanquish UHPLC system (Thermo Fisher, Germany) coupled with an Orbitrap Q Exactive™ HF mass spectrometer or Orbitrap Q Exactive™ HF-X mass spectrometer (Thermo Fisher, Germany) in Novogene Co., Ltd. (Beijing, China). Samples were injected onto a Hypersil Gold column (100 × 2.1 mm, 1.9 μm) using a 12-min linear gradient at a flow rate of 0.2 mL/min. The eluents for the positive and negative polarity modes were eluent A (0.1% FA in Water) and eluent B (Methanol). The solvent gradient was set as follows: 2% B, 1.5 min; 2-85% B, 3 min; 85-100% B, 10 min; 100-2% B, 10.1 min; 2% B, 12 min. Q Exactive™ HF mass spectrometer was operated in positive/negative polarity mode with spray voltage of 3.5 kV, capillary temperature of 320 °C, sheath gas flow rate of 35 psi and aux gas flow rate of 10 L/min, S-lens RF level of 60, Aux gas heater temperature of 350 °C.

### 2.12 Measurement of testicular antioxidant capacity and lipid peroxidation

The activities of antioxidant enzymes and the levels of lipid peroxidation in testicular tissues were determined using specialized biochemical assay kits (Solarbio Science & Technology, Beijing, China). Briefly, frozen testis samples were weighed and homogenized in cold extraction buffer or physiological saline (1:9, w/v). The homogenates were centrifuged at 12,000×*g* for 10 min at 4 °C, and the resulting supernatants were collected for subsequent analysis. The protein concentration of each sample was determined using a BCA Protein Assay Kit to normalize the biochemical parameters. Superoxide dismutase (SOD) activity (BC5165) was measured via the xanthine oxidase method, which utilizes the WST-8 reagent to detect superoxide radicals. Catalase (CAT) activity (BC0205) was evaluated by monitoring the decrease in absorbance at 240 nm resulting from the decomposition of H_2_O_2_. To assess lipid peroxidation, malondialdehyde (MDA) content (BC0020) was initially determined using the thiobarbituric acid (TBA) reactive method. In strict accordance with contemporary methodological guidelines regarding the non-specific nature of TBA reactivity, these results are expressed as thiobarbituric acid reactive substances (TBARS). All data were normalized to the total protein content and expressed as units per milligram of protein (U/mg prot) or nanomoles per milligram of protein (nmol/mg prot).

### 2.13 Evaluation of general intracellular oxidative stress

The relative levels of general intracellular oxidative stress were assessed using the 2′,7′-dichlorodihydrofluorescein diacetate (DCFH-DA) fluorescent probe (S0033 M, Beyotime Biotechnology, Shanghai, China). Briefly, following the designated treatments, TM3 Leydig cells were incubated with 10 μM DCFH-DA in serum-free medium for 20 min at 37 °C in a light-protected environment. The DCF fluorescence intensity, which serves as a non-specific surrogate marker for the intracellular redox state rather than a direct measurement of specific reactive oxygen species, was captured using a fluorescence microscope. To ensure quantitative comparability, all images were acquired using consistent exposure times and detector gain settings across all experimental groups. For quantitative analysis, at least 10,000 events were recorded via flow cytometry, and the mean fluorescence intensity (MFI) was calculated using FlowJo software.

### 2.14 Western blot analysis

Cells were collected and lysed with RIPA buffer supplemented with protease and phosphatase inhibitors. Clarified lysates were quantified by BCA assay, mixed with Laemmli buffer, and denatured (95 °C, 5 min). Equal protein (20-30 μg) was resolved by SDS–PAGE (8-12%) and transferred to methanol-activated PVDF membranes. Membranes were blocked in 5% BSA for 1 h at room temperature, then incubated overnight at 4 °C with primary antibodies diluted in blocking buffer (details in Supplementary Table 1). After washing with TBST buffer, secondary antibodies were used at 1:2000 dilutions for 1h at room temperature. Bands were developed with enhanced chemiluminescence and imaged on a chemiluminescence system with exposures constrained to the linear range.

### 2.15 Mitochondrial membrane potential (ΔΨm) assay

The mitochondrial membrane potential (ΔΨm) of TM3 Leydig cells was assessed using a ratiometric JC-1 Assay Kit (C2003S; Beyotime Biotechnology, Shanghai, China). Briefly, a 1X JC-1 working solution was freshly prepared by diluting the 200x stock in staining buffer (1:200, v/v). Following the designated treatments, cells were incubated with the JC-1 working solution for 20 min at 37 °C in the dark. After two washes with assay buffer, the cells were maintained in phenol-red-free buffer at 37 °C for live-cell imaging. To ensure quantitative comparability, all images were acquired using a confocal laser scanning microscope with identical laser power, gain, and offset settings across all experimental groups. JC-1 monomers were excited at 488 nm (emission: 510-550 nm), and J-aggregates were excited at 552/561 nm (emission: 570-620 nm). Sequential acquisition was employed to eliminate potential fluorescence bleed-through between channels. At least five random fields were captured from each of the three independent biological replicates. For quantification, the per-cell red-to-green fluorescence intensity ratio was calculated after background subtraction using ImageJ software. Field-averaged means were used as the unit of analysis and normalized to the control group.

### 2.16 Assessment of mitochondrial membrane potential (ΔΨm) via TMRE

Mitochondrial membrane potential (ΔΨm) was quantitatively assessed using tetramethylrhodamine ethyl ester (TMRE; T669; Thermo Fisher Scientific, Waltham, MA, USA). As a Nernstian indicator, TMRE was employed to provide a thermodynamically robust and sensitive estimation of ΔΨm, adhering to the updated methodological guidelines for mitochondrial bioenergetics [41]. Briefly, following the designated treatments, TM3 Leydig cells were incubated with 200 nM TMRE in serum-free medium for 20 min at 37 °C in a light-protected environment. After incubation, cells were washed twice with pre-warmed PBS to eliminate non-sequestered probe and minimize extracellular background. Fluorescence intensity was immediately quantified via flow cytometry using an excitation wavelength of 561 nm and an emission filter of 585 nm (PE channel). For each sample, a minimum of 10,000 events were recorded. Data analysis was performed using FlowJo software (version 10.8.1; Ashland, OR, USA) to determine the mean fluorescence intensity.

### 2.17 Mitochondrial morphological evaluation via Mito-Tracker staining

Mitochondrial morphology and functional distribution in TM3 Leydig cells were evaluated using the Mito-Tracker™ Red CMXRos fluorescent probe (C1049B; Beyotime Biotechnology, Shanghai, China). As a cationic, lipid-soluble fluorophore, CMXRos sequestered within active mitochondria in a membrane potential-dependent manner, facilitating the direct observation of mitochondrial fluorescence changes and structural integrity. Briefly, following the designated treatments, cells were incubated with 100 nM CMXRos in serum-free medium for 30 min at 37 °C in a light-protected environment. To ensure the acquisition of high-quality signals while minimizing non-specific background, cells were rinsed twice with pre-warmed, phenol-red-free medium. Live-cell imaging was performed immediately using a Nikon A1R + confocal laser scanning microscope equipped with a 60X oil-immersion objective and a temperature-controlled stage set at 37 °C. The mean fluorescence intensity (MFI) and morphological parameters were quantified using ImageJ software.

### 2.18 Detection of mitochondrial superoxide production

Mitochondrial superoxide (O_2_^• -^) levels were evaluated using the MitoSOX™ Green mitochondrial superoxide indicator [42] (M36005; Thermo Fisher Scientific, Waltham, MA, USA). Briefly, TM3 cells were incubated with 5 μM MitoSOX Green working solution for 15 min at 37 °C in a light-protected environment. After incubation, cells were washed three times with pre-warmed staining buffer (BD Biosciences) to remove residual extracellular dye. For quantitative analysis, the mean fluorescence intensity (MFI) was determined via flow cytometry, utilizing an excitation wavelength of 488 nm and an emission filter of 510 nm (FITC channel). Additionally, visual confirmation of mitochondrial superoxide(O_2_^• -^) localization was obtained using a Nikon A1R + confocal laser scanning microscope (Nikon, Tokyo, Japan). Images were captured using a 60X oil-immersion objective with consistent acquisition settings across groups. Data were analyzed using ImageJ, and the results were expressed as the fold change in MFI relative to the control group.

### 2.19 Pregnenolone ELISA in TM3 cell supernatants

Pregnenolone in conditioned media from TM3 Leydig cells was quantified by competitive ELISA (EU0380; FineTest, Wuhan, China) following the manufacturer's protocol with minor adaptations. After treatments, supernatants were collected, clarified (1000×*g*, 10 min, 4 °C), aliquoted, and stored at −80 °C (single freeze–thaw). Thawed samples were assayed undiluted in duplicate. Briefly, 50 μL standards or samples were added to antibody-precoated wells followed by 50 μL biotinylated detection antibody and incubated 60 min at 37 °C. Plates were washed 3 × , incubated with 100 μL HRP–streptavidin (30 min, 37 °C), washed, developed with 90 μL TMB (15 min, dark, 37 °C), stopped with 50 μL stop solution, and read at 450 nm (with 630 nm reference when available).An 8-point kit standard curve was fit by four-parameter logistic (4 PL) regression; sample concentrations were interpolated (dilution-corrected) and reported as ng/mL supernatant. Assay precision was monitored (intra-assay CV < 10%); samples outside the dynamic range were re-assayed after appropriate dilution. Results reflect ≥3 independent experiments.

### 2.20 Measurement of dihydrotestosterone (DHT) levels

The concentration of dihydrotestosterone (DHT) in testicular tissues was quantified using a commercial DHT ELISA Kit (D751011; Sangon Biotech, Shanghai, China) according to the manufacturer's instructions. Briefly, freshly collected testicular tissues were rinsed with ice-cold PBS (0.01 M, pH 7.4) to remove residual blood, weighed, and minced into small pieces. The minced tissues were homogenized in ice-cold PBS (recommended weight-to-volume ratio of 1:9) containing protease inhibitors using a glass homogenizer on ice. The homogenate was further disrupted by sonication and then centrifuged at 5000×*g* for 5 min. The resulting supernatant was collected for subsequent assay. The OD values were measured at 450 nm using a microplate reader. A standard curve was generated by plotting the absorbance (OD values) against the known concentrations of DHT standards and fitted using a four-parameter logistic (4 PL) regression model. Samples with OD values below the detection limit of the standard curve were appropriately diluted and re-assayed; the calculated concentrations were then multiplied by the corresponding dilution factor.

### 2.21 Human testicular tissue collection

Human testicular tissues were obtained from obese individuals (body mass index [BMI] ≥30 kg/m^2^; n = 4) diagnosed with oligoasthenoteratozoospermia (OAT), who had not history of prior hormonal intervention. These specimens were collected as residual tissues following therapeutic testicular sperm extraction (TESE). Upon procurement, the testicular tissues were immediately dissected into 2 mm fragments using sterile micro-scissors and positioned on 1% agarose gel platforms. The fragments were maintained in specialized spermatogonial stem cell culture medium (GCM-H191; Procell Life Science & Technology Co., Ltd., Wuhan, China) at 34 °C in a humidified atmosphere containing 5% CO_2_. The culture medium was replenished every 48h. All participants provided written informed consent for the voluntary donation of biological samples for research purposes. This study was conducted in strict accordance with the ethical principles outlined in the Declaration of Helsinki and was formally approved by the Ethics Committee of the Third Affiliated Hospital of Guangzhou Medical University (Approval No. 2025025). All procedures were performed in compliance with institutional guidelines and applicable national regulations for human clinical research.

### 2.22 Assessment of testicular general oxidative fluorescence index

The relative levels of general oxidative stress in testicular tissues were quantified using a non-specific oxidative fluorescence assay kit (BB-470538; BestBio Biotechnology, Shanghai, China) following the manufacturer's instructions. Briefly, fresh testes were homogenized on ice in the kit extraction buffer and clarified at 4 °C. Supernatants were incubated with the D01 working solution for 30 min at 37 °C in the dark. Fluorescence was recorded on a multimode reader (Ex 488 nm/Em 530 nm) and blank-subtracted using dye-only wells run on the same plate. To ensure quantitative accuracy, sample protein concentrations were determined using a BCA Protein Assay Kit (23227; Thermo Fisher Scientific, Waltham, MA, USA), and results were expressed as relative fluorescence units per milligram of protein (RFU/mg prot). In adherence to contemporary redox research guidelines, we acknowledge that the D01 probe serves as a general indicator of the intracellular redox state rather than a specific reactive oxygen species. Consequently, these data were interpreted as a non-specific oxidative fluorescence index and were utilized as supportive evidence when concordant with the orthogonal redox and mitochondrial readouts presented elsewhere in this study. All measurements were performed in technical duplicates across at least three independent biological replicates. Prior to statistical analysis, the normality of the data distribution was rigorously verified using the Shapiro-Wilk test.

### 2.23 Statistical analysis

All data are shown as mean ± standard error of the mean (SEM). All data were tested using the Shapiro–Wilk normality test and were normally distributed. Comparisons between the two groups were analyzed using Mann–Whitney *U* test. Single-factor multiple group comparisons were performed using one-way analysis of variance (ANOVA) test followed by Tukey's test for normally distributed data or Welch ANOVA followed by Tamhane's T2 test for non-normally distributed data. Two-factor multiple group comparisons were performed using two-way ANOVA followed by Tukey's test. Statistical significance was defined as *p* < 0.05. Bioinformatic analyses were performed using the OmicStudio tools available at https://www.omicstudio.cn/tool. Statistical analyses were performed using GraphPad Prism 9.5.1.

## 3 Results

### 3.1 Chronic high-fat diet progressively impairs testicular architecture and sperm quality

**Fig. 1:**
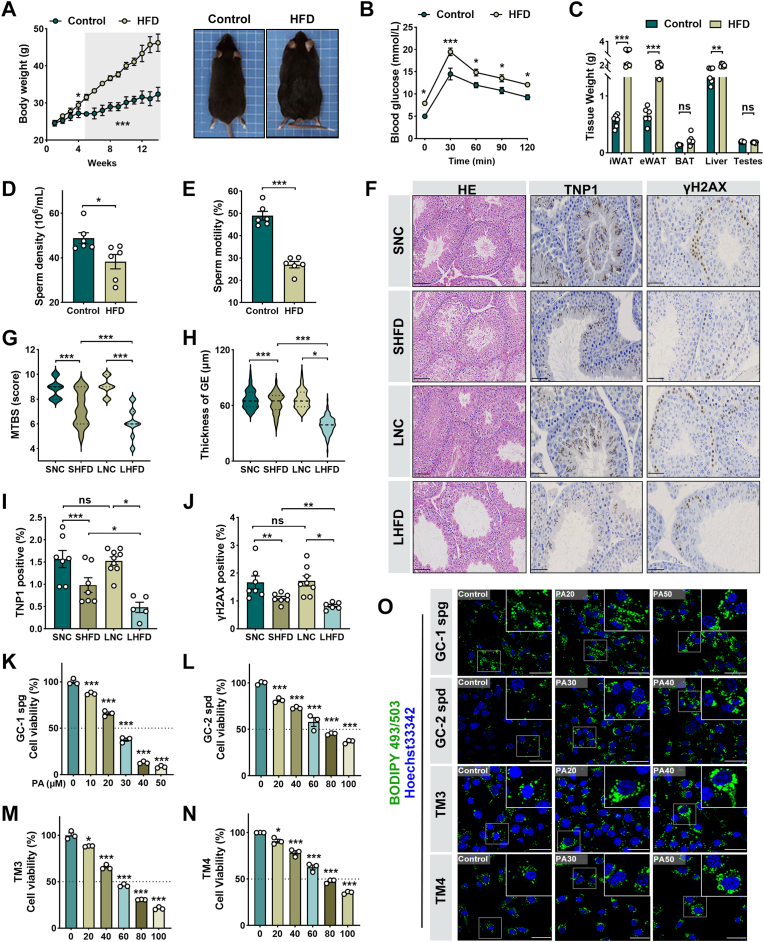
**Chronic high-fat diet progressively impairs testicular architecture and sperm quality.** (A) Longitudinal body weight changes in control and HFD-fed mice over a 14-week period (left), and representative gross morphology showcasing significant obesity in the HFD group (right). *n* = 6. (B) Oral glucose tolerance test (OGTT) profiles demonstrating impaired glucose homeostasis in HFD mice. (C) Adipose and organ weights, including epididymal fat, inguinal fat, liver, brown adipose tissue (BAT), and testes, normalized to body weight. (D-E) Quantitative analysis of sperm concentration and total motility assessed by computer-assisted sperm analysis (CASA). (F) Representative H&E-stained sections of testicular tissues from SNC (Short-term Normal Control), SHFD (Short-term HFD), LNC (Long-term Normal Control), and LHFD (Long-term HFD) groups. scale bar = 50 μm. (G-H) Representative immunohistochemical (IHC) staining, morphometric quantification of the Mean Tubular Biopsy Score (MTBS) and germinal epithelium (GE) thickness, reflecting the degree of seminiferous tubule degeneration. (I-J) Quantification of Transition Protein 1 (TNP1) and γH2AX in testicular sections. γH2AX serves as a marker for DNA double-strand breaks in meiotic spermatocytes. scale bar = 20 μm. (K–N) Cytotoxicity profiles of mouse testicular cell lines (GC-1 spg, GC-2 spd, TM3, and TM4) following exposure to varying concentrations of palmitate (PA) for 48 h, assessed via CCK-8 assay. (O) Representative fluorescence images of BODIPY™ 493/503 staining visualizing neutral lipid droplet (LD) accumulation in GC-1 spg, GC-2 spd, TM3, and TM4 cells under lipotoxic conditions. scale bar = 20 μm. Data are presented as mean ± SEM (*n* ≥ 3 independent biological replicates). The normality of the data was verified using the Shapiro-Wilk test. Statistical significance was determined by one-way ANOVA followed by Tukey's post-hoc test. ∗*P* < 0.05, ∗∗*P* < 0.01, ∗∗∗*P* < 0.001 indicate significant differences compared to the control group.

Chronic high-fat diet (HFD) feeding induced systemic obesity and progressively impaired male reproductive function. After 14 weeks, HFD-fed mice displayed a marked increase in body weight and glucose intolerance compared with chow-fed controls, confirming the successful establishment of metabolic stress (Fig. 1A–B). Interestingly, testis weight remained unchanged, yet reproductive performance was significantly compromised, as shown by reduced sperm concentration and motility measured by computer-assisted sperm analysis (Fig. 1C–E). These data suggest that testicular dysfunction can develop independently of gross testis atrophy. Histopathological analyses revealed striking alterations in the seminiferous epithelium of long-term HFD (LHFD) mice. Disorganized germ cell layers, reduced mature spermatids, and thinning of the germinal epithelium (GE) were observed, leading to significantly decreased mean testicular biopsy scores (MTBS) (Fig. 1F–H). Immunohistochemistry further confirmed reduced expression of TNP1 and γH2AX, markers of germ cell differentiation and genome integrity, indicating disrupted spermatogenesis and meiotic defects. To better assess the progressive effect of testicular injury, a short-term HFD (SHFD, 9 weeks) group was analyzed in parallel. SHFD mice showed only mild structural alterations and less pronounced spermatogenic impairment, whereas LHFD mice displayed more severe degeneration, supporting a progressive trajectory of reproductive decline under sustained metabolic stress (Fig. 1I–J). To mechanistically test whether lipid overload directly contributes to cytotoxicity, palmitic acid (PA)—a saturated fatty acid elevated in obesity—was applied to four testicular cell lines (GC-1 spg, GC-1 spd, TM3, and TM4). PA exposure caused prominent lipid droplet accumulation and dose-dependent loss of viability, thereby recapitulating the in vivo phenotype of lipotoxic stress (Fig. 1K–O; Fig. S1, Supporting Information). Collectively, these results establish that chronic HFD progressively damages the testis through a combination of systemic metabolic dysregulation and lipid-induced cellular toxicity.

### 3.2 HFD reprograms testicular metabolism and suppresses steroidogenesis

**Fig. 2:**
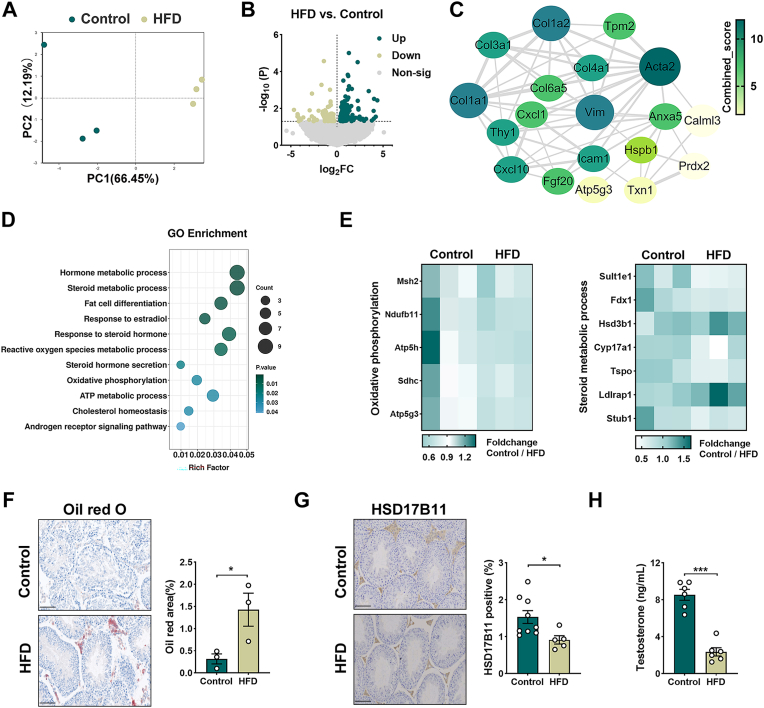
HFD reprograms testicular metabolism and suppresses steroidogenesis. (A) Partial least squares-discriminant analysis (PLS-DA) score plot illustrating the distinct metabolic trajectories and clustering of testicular profiles between the HFD and Control groups. (B) Volcano plot identifying differentially expressed genes (DEGs) based on the criteria of |log_2_FC| > 0 and *P* < 0.05 between the HFD and Control cohorts. (C) Topological visualization of the protein–protein interaction (PPI) network of the identified DEGs, constructed using the STRING database and visualized via Cytoscape to identify core functional modules. (D) Gene Ontology (GO) enrichment analysis categorizing the DEGs into specific biological processes, highlighting the impact of HFD on metabolic regulation. (E) Transcriptional heatmap depicting the expression patterns of key genes involved in steroid metabolism and oxidative phosphorylation (OXPHOS) pathways (*n* = 3 per group). (F) Representative images and morphometric quantification of Oil Red O-stained testicular sections, demonstrating excessive neutral lipid accumulation in HFD-treated mice. scale bar = 50 μm. (G) Representative immunohistochemical (IHC) images and semi-quantitative analysis of HSD17B11 protein expression within the testicular interstitium. scale bar = 50 μm. (H) Quantification of intratesticular testosterone levels, reflecting the functional state of Leydig cells. Data are presented as mean ± SEM (*n* = 6). The normality of the data distribution was rigorously confirmed using the Shapiro-Wilk test. Statistical significance was determined using one-way ANOVA followed by Tukey's post-hoc test. ∗*P* < 0.05, ∗∗*P* < 0.01, ∗∗∗*P* < 0.001 indicate significant differences compared to the control group.

To define the molecular pathways linking obesity-induced testicular dysfunction, we performed transcriptomic profiling of testes from mice fed HFD for 14 weeks. Principal component and volcano plot analyses revealed extensive transcriptional remodeling, and more than 300 genes were significantly altered compared with controls. (Fig. 2A–B). To prioritize central regulatory nodes among these DEGs, we constructed a protein–protein interaction (PPI) network and identified the top 20 hub genes (Fig. 2C). Acta2 was identified as the central hub gene, highlighting its essential role in driving adipose fibrosis—a key mechanism in obesity-related metabolic dysfunction. Col1a1 further supports extracellular matrix deposition, while Txn1 regulates oxidative stress in adipocytes. Other genes including Thy1 and Vim contribute to inflammation and cytoskeletal changes, corroborating the network's involvement in adipose remodeling and redox imbalance in obesity. Pathway enrichment analysis of DEGs further confirmed significant enrichment of steroid hormone biosynthesis, lipid metabolism, reactive oxygen species regulation, and ATP-linked mitochondrial pathways (Fig. 2D). Heatmap visualization highlighted coordinated downregulation of oxidative phosphorylation and steroidogenic enzymes genes, pointing to simultaneous suppression of endocrine and mitochondrial bioenergetic programs (Fig. 2E).

Given that the balance of steroid metabolism is essential for maintaining spermatogenesis, we next examined histological and biochemical correlates. Oil Red O staining revealed pronounced lipid accumulation in interstitial Leydig cell, suggesting that HFD-mediated lipid overload imposes direct lipotoxic stress on the steroidogenic compartment (Fig. 2F). Consistently, Leydig cell markers HSD17B11 was markedly downregulated, and intratesticular testosterone levels were significantly reduced in HFD-fed mice (Fig. 2G–H). Together, these results indicate that HFD reprograms the testicular environment by suppressing steroidogenic signaling network while promoting lipid deposition and lipotoxic injury, thereby creating a metabolic milieu unfavorable for spermatogenesis.

### 3.3 Progressive depletion of ergothioneine and lipid remodeling defined in the HFD testes

**Fig. 3:**
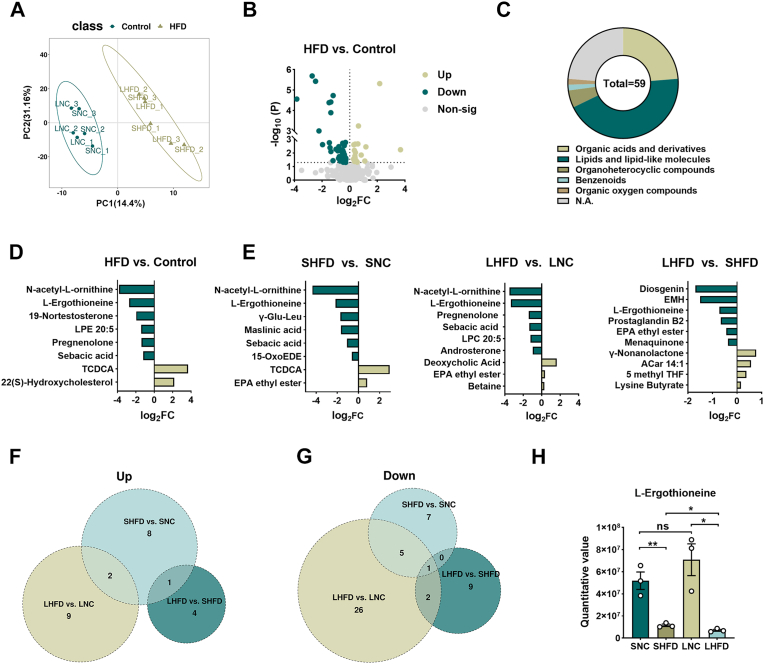
**Progressive depletion of ergothioneine and lipid remodeling defined in the HFD testes**. (A) Multivariate partial least squares-discriminant analysis (PLS-DA) score plot illustrating a distinct separation of metabolic phenotypes between the HFD and Control groups at different time points. (B) Volcano plot identifying significantly altered metabolites based on the criteria of variable importance in projection(VIP) > 1.0 and *P* < 0.05. (C) Chemical class distribution of the identified differential metabolites, highlighting major lipid and organic acid remodeling. (D) Differential abundance of top-ranked testicular metabolites, showing significantly upregulated and downregulated molecules across experimental cohorts. (E) Summary of the total number of differential metabolites identified through pairwise comparisons (SHFD vs. SNC; LHFD vs. LNC; and LHFD vs. SHFD), demonstrating the temporal metabolic shift. (F-G) Venn diagrams depicting intersectional analysis of shared and unique (F) upregulated and (G) downregulated metabolites among the indicated longitudinal comparisons. (H) Longitudinal quantification of l-Ergothioneine (ET) levels in testicular tissues across the SNC, SHFD, LNC, and LHFD groups, showcasing its progressive depletion during HFD-induced obesity. Data are presented as mean ± SEM (*n* = 6 biological replicates for metabolomics). The normality of the metabolic data was rigorously assessed using the Shapiro-Wilk test. For multiple group comparisons, one-way ANOVA followed by Tukey's post-hoc test was employed. ∗*P* < 0.05, ∗∗*P* < 0.01, ∗∗∗*P* < 0.001 versus the respective control group.

Given the transcriptomic evidence that HFD perturbs steroidogenic and metabolic pathways, we next performed untargeted LC-MS/MS metabolomics on testes from both short- and long-term HFD cohorts to validate these changes at the metabolite level. Orthogonal PLS-DA showed clear separation between control and HFD groups, indicating global metabolic disruption (Fig. 3A). A total of 59 significantly altered metabolites were identified, primarily classified as lipids, organic acids, and heterocyclic compounds (Fig. 3B–C). Among the discriminant metabolites, key steroidogenic intermediates—including pregnenolone and 19-nortestosterone—were consistently reduced, providing direct biochemical evidence of impaired androgen biosynthesis (Fig. 3D).

Time-course analyses further demonstrated cumulative metabolic perturbations, as both the number and magnitude of altered metabolites increased with HFD duration (24 in SHFD, 45 in LHFD), highlighting progressive deterioration of the metabolic landscape (Fig. 3E). Venn analysis revealed that several metabolites showed consistent alterations across groups: N-acetyl-L-ornithine and sebacic acid were significantly downregulated in both SHFD and LHFD testes, while eicosapentaenoic acid ethyl ester and 22(S)-hydroxycholesterol were upregulated in both cohorts, indicating early-onset shifts in amino acid and lipid metabolism. By contrast, pregnenolone and androsterone were significantly reduced only in the LHFD group, suggesting that prolonged HFD exposure is required to impair steroidogenic capacity. Strikingly, ergothioneine (ET) was the only metabolite consistently altered across all three pairwise comparisons, with levels declining steadily as HFD duration increased (Fig. 3F–H). Given ET's potent antioxidant and anti-inflammatory properties, this unique temporal depletion of ET underscores its potential as a sensitive biomarker of cumulative redox stress and progressive testicular dysfunction.

### 3.4 ET supplementation restores spermatogenesis and testicular architecture under HFD stress

**Fig. 4:**
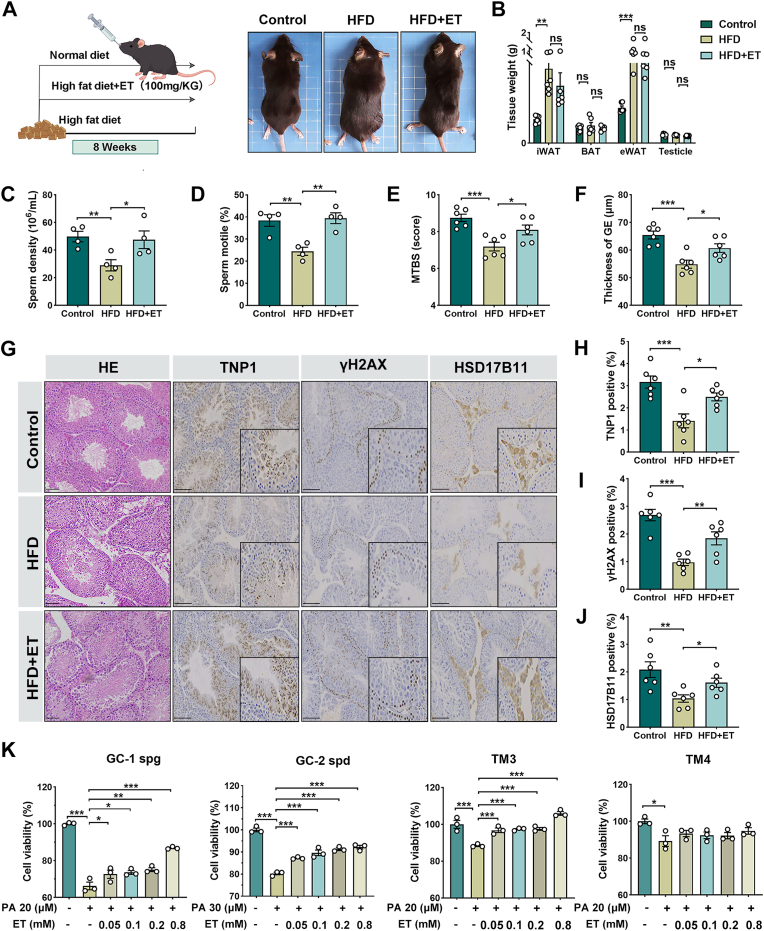
ET supplementation restores spermatogenesis and testicular architecture under HFD stress. (A) Schematic representation of the experimental therapeutic regimen: mice were randomly allocated into three cohorts (*n* = 6): Control (standard chow), HFD (60% kcal fat), and HFD + ET (HFD supplemented with 100 mg/kg/day ET via oral gavage) for an 8-week period. (B) Morphometric organ weight measurements, including inguinal white adipose tissue (iWAT), epididymal white adipose tissue (eWAT), scapular brown adipose tissue (BAT), and the testes-to-body weight ratio across experimental groups. (C-D) Quantitative assessment of sperm concentration and total motility, showcasing the restorative effect of ET on sperm quality. (E-F) Histomorphometric quantification of the Mean Tubular Biopsy Score (MTBS) and germinal epithelium (GE) thickness in H&E-stained testicular sections. (G-J) Representative H&E-stained histoarchitecture and immunohistochemical (IHC) localization of Transition Protein 1 (TNP1), γH2AX, and HSD17B11 in testicular tissues. γH2AX was utilized to monitor DNA double-strand breaks during meiotic prophase. scale bar = 50 μm. (K) Cell viability profiles of mouse testicular cell lines (GC-1 spg, GC-2 spd, TM3, and TM4) challenged with palmitate (PA) in the presence or absence of varying concentrations of ET (0.05, 0.1, 0.2, or 0.8 mM) for 48 h, determined via CCK-8 assay (*n* = 3). Data are presented as mean ± SEM. The normality of the data distribution was rigorously verified using the Shapiro-Wilk test. Statistical significance was determined by one-way ANOVA followed by Tukey's post-hoc test. ∗*P* < 0.05, ∗∗*P* < 0.01, ∗∗∗*P* < 0.001 indicate significant differences compared to the control group.

To further clarify the functional role of ET in the testicular microenvironment, HFD-induced obese male mice were treated with ET by oral gavage for 8 weeks (Fig. 4A). ET administration did not significantly alter body weight or adipose tissue mass (Fig. 4B). However, sperm density and motility were markedly improved, demonstrating functional recovery of spermatogenesis (Fig. 4C–D). Histological analyses further revealed that ET supplementation restored the orderly architecture of seminiferous epithelium, increased MTBS, and thickened the germinal epithelium compared with HFD controls (Fig. 4E–G). Immunohistochemistry staining further demonstrated upregulation of spermatogenic and steroidogenic markers, including TNP1, γH2AX, and HSD17B11, signifying improved germ cell development and enhanced Leydig cell activity (Fig. 4H–J). Consistent with these in vivo findings, in vitro assays showed that ET markedly improved the survival of PA-challenged testicular cell lines, with the most pronounced protective effects in TM3 Leydig cells (Fig. 4K). This pattern highlights Leydig cells as a key cellular target of ET action. Collectively, these results demonstrate that ET supplementation preserves both the structural integrity and functional output of the testis under metabolic stress. By mitigating spermatogenic damage and enhancing Leydig cell resilience, ET emerges as a promising protective agent in obesity-associated male infertility.

### 3.5 ET realigns transcriptome-metabolome interactions to restore steroidogenic and antioxidant balance in HFD testes

**Fig. 5:**
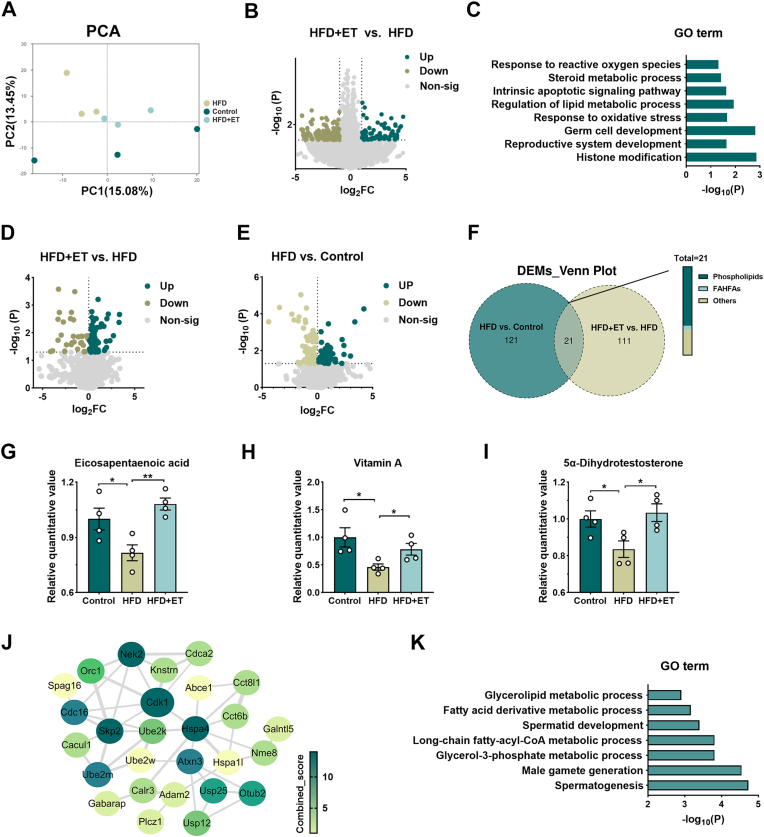
**ET realigns transcriptome-metabolome interactions to restore steroidogenic and antioxidant balance in HFD testes**. (A) Principal Component Analysis (PCA) illustrating the distinct clustering of testicular transcriptomic profiles among the Control, HFD, and HFD + ET groups based on RNA-seq data (*n* = 3 per group). (B) Volcano plots identifying significantly differentially expressed genes (DEGs) based on the criteria of |log_2_FC| > 1, *P* < 0.05 for the indicated pairwise comparisons (HFD vs. Control and HFD + ET vs. HFD). (C) Comparative Gene Ontology (GO) enrichment analysis of the identified DEGs, highlighting restored biological pathways in the HFD + ET group. (D–E) Volcano plots characterizing significantly altered testicular metabolites between the HFD + ET and HFD cohorts, indicating the metabolic recovery induced by ET. (F) Venn diagram illustrating the intersection of differential metabolites to identify overlapping metabolic signatures reversed by ET supplementation. (G-I) Relative abundance of keystone testicular metabolites, including eicosapentaenoic acid (EPA), vitamin A, and 5α-dihydrotestosterone (DHT), reflecting restored lipid and hormonal balance. (J) Integrative metabolite–gene correlation network (Pearson r > 0.8) identifying hub genes and their associated metabolic nodes, visualized via Cytoscape to uncover keystone regulatory interactions. (K) Functional GO enrichment analysis of the correlated targets, implicating core processes such as spermatogenesis, male gamete generation, and lipid metabolic regulation in the therapeutic mechanism of ET. Data are presented as mean ± SEM. The normality of both transcriptomic and metabolic datasets was verified using the Shapiro-Wilk test. For multiple group comparisons, one-way ANOVA followed by Tukey's post-hoc test was performed. ∗*P* < 0.05, ∗∗*P* < 0.01, ∗∗∗*P* < 0.001 versus the HFD group (or as indicated in the panels).

To delineate the molecular basis of ET protection, we performed integrative transcriptomic-metabolomic profiling of testes. PCA profiling demonstrated that ET supplementation shifted the HFD-altered transcriptomic landscape toward that of controls (Fig. 5A). Compared with HFD, more than 1000 differentially expressed genes were identified, enriched in pathways related to steroid metabolism, oxidative stress response, lipid regulation, and germ-cell development (Fig. 5B–C). Consistent with these transcriptomic changes, untargeted LC-MS/MS showed reversal of key HFD-driven metabolic disturbances (Fig. 5D–E). Multi-layer comparisons identified 21 intersected metabolites, 14 of which were phospholipids, indicating lipid-centric remodeling as a major axis of ET action (Fig. 5F). Importantly, ET enriched metabolites linked to antioxidant and androgenic activity, including vitamin A, eicosapentaenoic acid (EPA), and 5α-dihydrotestosterone (DHT) (Fig. 5G–I), directly supporting improved steroidogenic and redox capacity. Integration of transcriptomic and metabolomic datasets (Pearson r > 0.8) identified a subset of ET-associated genes that exhibited strong intercorrelations (Fig. 5J). Functional enrichment analysis revealed that these genes were predominantly involved in lipid metabolism, spermatogenesis, and male gamete generation (Fig. 5K). This integrative, systems-level approach indicates that ET supplementation not only restores key metabolites but also activates coordinated gene networks, thereby re-establishing steroidogenic and antioxidant balance in the testis and ultimately promoting spermatogenic recovery.

### 3.6 ET preserves mitochondrial integrity and antioxidant defenses in HFD-impaired testes

**Fig. 6:**
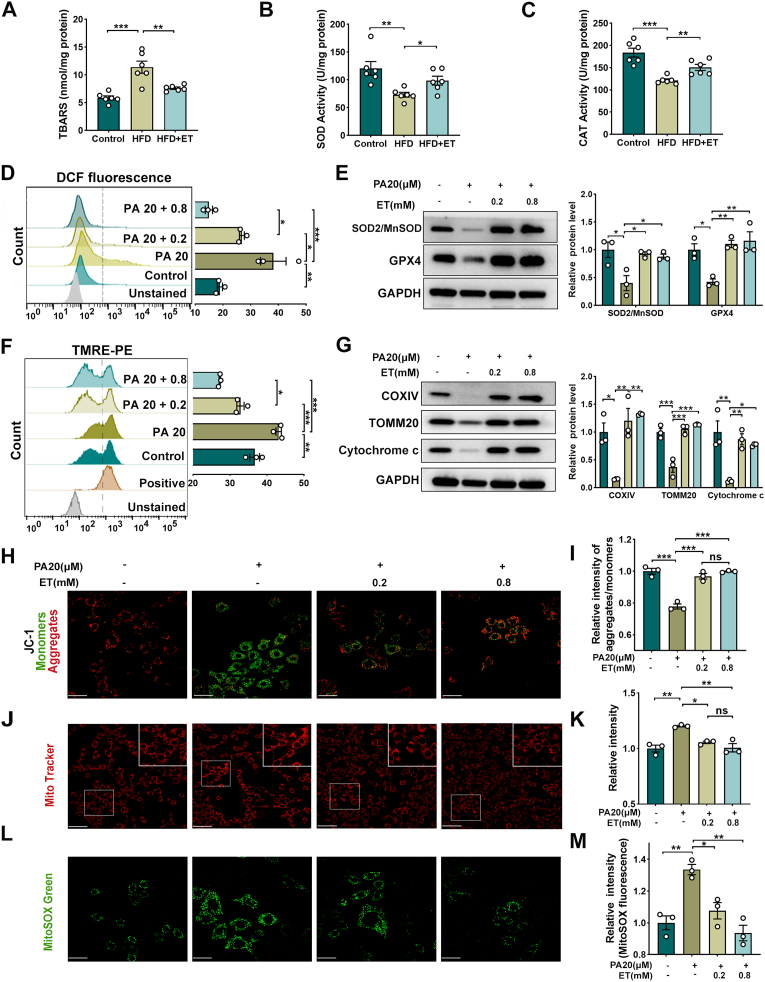
ET preserves mitochondrial integrity and antioxidant defenses in HFD-impaired testes. (A) Concentrations of thiobarbituric acid reactive substances (TBARS) in testicular tissues, utilized as a surrogate marker for lipid peroxidation. (B–C) Activities of enzymatic antioxidants, including superoxide dismutase (SOD) and catalase (CAT), normalized to total protein content. (*n* = 6 per group). (D) Representative flow cytometry histograms and quantitative analysis of DCF fluorescence intensity in TM3 cells challenged with palmitate (PA, 20 μM) and treated with ET (0.2, 0.8 mM). DCF fluorescence reflects the general intracellular oxidative stress. (E) Western blot analysis and corresponding quantification of mitochondrial antioxidant enzymes, SOD2 and GPX4. GAPDH served as the loading control. (F) Representative flow cytometry plots and quantitative analysis of TMRE fluorescence (MFI). TMRE was employed as a Nernstian indicator to monitor the mitochondrial membrane potential (*ΔΨm).* (G) Western blot analysis of mitochondrial structural and biogenetic markers, including COX IV, TOMM20, and Cytochrome *c*, following PA and ET treatments. (H-M) Representative confocal laser scanning microscopy images of TM3 cells stained with: MitoSOX Red: A specific indicator for mitochondrial superoxide(O_2_^•-^). production. JC-1: Visualizing *ΔΨm* through the transition from red J-aggregates to green monomers. Mito-Tracker Red CMXRos: Specifically labeling active mitochondria to verify organelle morphology and localization. scale bar = 50 μm. Data are presented as mean ± SEM from at least three independent biological replicates (*n* = 3). Statistical significance was evaluated using one-way ANOVA followed by Tukey's post-hoc test; ∗*P* < 0.05, ∗∗*P* < 0.01, ∗∗∗*P* < 0.001 indicate significant differences compared to the control group.

Building on the integrative omics analysis that identified ET as a key regulator of redox and metabolic homeostasis, we investigated whether ET directly protects against HFD-induced oxidative damage and mitochondrial dysfunction. Given that oxidative stress is a hallmark of male subfertility [34,35,[43], [44], [45]], we systematically evaluated lipid peroxidation, enzymatic antioxidant capacity, and mitochondrial homeostatic markers both in vivo and in TM3 Leydig cells. In testicular tissues, HFD significantly exacerbated lipid peroxidation, as evidenced by the accumulation of thiobarbituric acid reactive substances (TBARS), a surrogate marker for systemic oxidative damage (Fig. 6A). This was accompanied by a marked suppression in the activities of core antioxidant enzymes, specifically SOD and CAT. ET supplementation effectively mitigated these pathological changes, lowering TBARS concentrations and restoring SOD and CAT activities to near-basal levels (Fig. 6B and C). To further elucidate the cellular mechanisms, TM3 cells were challenged with palmitate (PA) to mimic lipotoxic stress. Flow cytometric analysis of DCF fluorescence revealed that PA triggered a robust increase in general intracellular oxidative stress, while ET treatment induced a concentration-dependent leftward shift in the fluorescence peak, reflecting a restoration of the intracellular redox state (Fig. 6D). At the protein level, ET significantly upregulated the mitochondrial antioxidant enzymes SOD2 and GPX4, reinforcing the cellular defense against peroxide-mediated injury (Fig. 6E). Recognizing the limitations of single-probe assays, we employed a multi-dimensional approach to assess mitochondrial functional integrity. Quantitative TMRE flow cytometry—a Nernstian indicator of mitochondrial membrane potential (*ΔΨm*)—demonstrated that ET prevented the PA-induced collapse of *ΔΨm* (Fig. 6F). These functional improvements were underpinned by the fortified expression of mitochondrial structural and biogenetic markers, including COX IV, cytochrome *c*, and TOMM20 (Fig. 6G). To pinpoint the source of oxidative stress, we utilized MitoSOX Green, a specific indicator for mitochondrial superoxide (O_2_^•-^). Confocal imaging (using MitoSOX, JC-1, and Mito-Tracker Red CMXRos) provided visual confirmation that ET attenuated mitochondrial O_2_^•-^ overproduction and maintained polarized mitochondrial networks (Fig. 6H–M). Taken together, these data suggest that ET alleviates HFD-induced testicular damage by augmenting antioxidant capacity and preserving mitochondrial structural and functional integrity, thereby fostering an optimal microenvironment for sustained steroidogenesis and spermatogenesis.

### 3.7 ET rescues testosterone biosynthesis from lipotoxic stress by reinstating PKA-CREB-StAR signaling

**Fig. 7:**
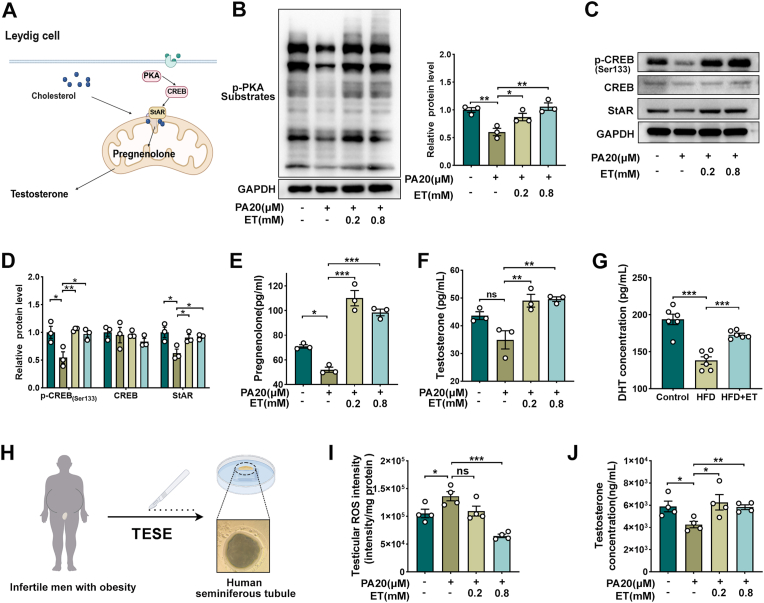
**ET rescues testosterone biosynthesis from lipotoxic stress by reinstating PKA-CREB-StAR signaling.** (A) Schematic illustration of the canonical steroidogenic pathway in Leydig cells, highlighting the key enzymes and intermediates from cholesterol to testosterone. (B-D) Representative immunoblotting images and densitometric quantification of phosphorylated PKA (*p*-PKA), *p*-CREB (Ser133), total CREB, and StAR protein abundance in TM3 Leydig cells challenged with palmitate (PA) in the presence or absence of ET. GAPDH was utilized as the internal loading control. (E-F) Quantitative analysis of pregnenolone and testosterone secretion levels in the culture supernatant of TM3 cells via Enzyme-Linked Immunosorbent Assay (ELISA), demonstrating the functional rescue of steroidogenesis by ET. (G) ELISA-based quantification of intratesticular 5α-dihydrotestosterone (DHT) levels in the Control, HFD, and HFD + ET mouse cohorts. (H) Schematic workflow of the ex vivo organ culture system using human seminiferous tubules. Specimens were obtained via therapeutic testicular sperm extraction (TESE) from obese patients presenting with oligoasthenoteratozoospermia. (I) Quantification of mitochondrial ROS levels in human seminiferous tubules treated with PA and ET. (J) Functional assessment of testosterone secretion in the human ex vivo tubule culture system following PA challenge and ET intervention. Data are presented as means ± SEM (*n* = 3). The normality of all datasets, including Western blot densitometry and ELISA measurements, was rigorously verified using the Shapiro-Wilk test. Statistical significance was determined by one-way ANOVA followed by Tukey's post-hoc test. ∗*P* < 0.05, ∗∗*P* < 0.01, ∗∗∗*P* < 0.001 versus the respective stress group (PA).

Having established that ET preserves mitochondrial integrity—a prerequisite for steroid hormone synthesis—we next sought to further elucidate its role in Leydig-cell steroidogenesis under lipotoxic stress. To this end, androgen output was evaluated across in vivo, in vitro, and ex vivo systems, with a focus on the canonical PKA-CREB-StAR signaling cascade that governs cholesterol transport into mitochondria, the rate-limiting step for testosterone production (Fig. 7A). At the signaling level, PA exposure inhibited phosphorylation of PKA and CREB and downregulated StAR expression (Fig. 7B–D), leading to reduced pregnenolone and testosterone production (Fig. 7E–F). This blockade at the cholesterol import step effectively impaired the steroidogenic cascade. ET supplementation effectively restored phosphorylation of PKA and CREB, normalized StAR levels, resulting in increased pregnenolone and testosterone output, confirming re-engagement of the PKA-CREB-StAR axis. Consistently, in vivo analysis showed that HFD suppressed intratesticular DHT, whereas ET supplementation reinstated its levels, further corroborating restoration of androgen biosynthesis (Fig. 7G). To assess potential translational relevance, we extended these findings to ex vivo human seminiferous tubule cultures from obese infertile men (Fig. 7H). While PA exposure provoked substantial mitochondrial oxidative damage and inhibited testosterone secretion, ET supplementation effectively neutralized the intracellular oxidative index and enhanced the steroidogenic output of the testis (Fig. 7I and J). Together, these results demonstrate that ET preserves Leydig-cell steroidogenic capacity by reactivating the PKA-CREB-StAR signaling axis in parallel with stabilizing mitochondrial redox balance. These dual protective actions provide a mechanistic foundation for future causal testing of this pathway in obesity-associated male infertility.

## 4 Discussion

This study provides a comprehensive characterization of how obesity disrupts testicular metabolism and how supplementation with ergothioneine (ET) restores reproductive function. Using an integrative framework that combined metabolomics, transcriptomics, in vivo mouse models, and ex vivo human seminiferous tubule cultures, we show that chronic high-fat diet (HFD) feeding leads to progressive spermatogenic dysfunction driven by redox imbalance, suppression of steroidogenesis, and mitochondrial impairment. Importantly, ET supplementation not only restores sperm quality and testicular architecture but also re-engages the PKA-CREB-StAR axis and stabilizes mitochondrial redox homeostasis. These findings highlight ET as both a mechanistic probe and a potential therapeutic adjunct for obesity-associated male infertility.

Obesity-related impairment of testicular steroidogenesis has been consistently reported in both clinical and experimental studies [12,46,47]. Previous work has largely attributed hypogonadism in obese males to systemic factors, including increased aromatase activity in adipose tissue, altered hypothalamic-pituitary-gonadal (HPG) axis signaling [48,49], insulin resistance, and chronic low-grade inflammation. In rodent models, HFD feeding reduces circulating testosterone levels and downregulates key steroidogenic enzymes such as StAR, Cyp11a1, and Hsd3b1, while in humans, obesity is strongly associated with reduced total and free testosterone levels. However, the extent to which obesity induces testis-intrinsic metabolic remodeling—independent of systemic endocrine feedback—has remained insufficiently explored.

At the molecular level, our integrated transcriptomic and metabolomic analyses provide direct tissue-level evidence that obesity profoundly remodels the testicular metabolic landscape. We observed coordinated downregulation of steroidogenic gene networks and oxidative phosphorylation pathways, accompanied by depletion of key steroidogenic intermediates, including pregnenolone and its precursors. These findings extend prior observations by demonstrating that obesity compromises androgen biosynthesis not only through endocrine dysregulation but also via intrinsic mitochondrial and metabolic dysfunction within the testis. In parallel, the accumulation of interstitial lipids and altered lipid species suggests a lipotoxic microenvironment that may further exacerbate Leydig and germ cell dysfunction, consistent with emerging concepts of lipid-induced mitochondrial stress in steroidogenic tissues.

A particularly novel observation is the progressive decline of testicular ET content with increasing HFD duration. ET is a unique redox-active thiol with well-documented cytoprotective properties and the ability to accumulate selectively in metabolically active and oxidation-prone tissues. Previous studies have reported high ET concentrations in erythrocytes, liver, kidney, semen, and ocular tissues, with levels ranging from 100 μmol/L to 2 mmol/L [[50], [51], [52], [53], [54], [55], [56]]. Although its physiological role under basal conditions remains incompletely defined, ET is increasingly recognized as an integral component of the endogenous antioxidant defense system [52,[57], [58], [59]]. Notably, circulating ET levels decline with age and in several diseases, including gastrointestinal disorders [60], neurodegenerative diseases [61,62], cardiovascular diseases [63], diabetes [64], and chronic kidney diseases [65,66]. However, its role in obesity-associated male reproductive dysfunction had not been examined. Our data indicate that ET depletion represents a previously unrecognized feature of testicular metabolic stress in obesity. This observation provides a mechanistic bridge between redox imbalance and impaired steroidogenesis, suggesting that loss of endogenous antioxidant buffering capacity may sensitize Leydig cells to lipotoxic and mitochondrial insults. In this context, ET emerges not only as a biomarker candidate but also as a functional determinant of testicular resilience under metabolic stress.

Mechanistic experiments further show that ET directly counteracts lipotoxic injury in Leydig cells. ET restores testosterone synthesis by re-engaging the PKA-CREB-StAR signaling cascade, which governs cholesterol transport into mitochondria—a recognized rate-limiting step in steroidogenesis. By normalizing pregnenolone production and upregulating steroidogenic enzymes, ET relieves a critical bottleneck in androgen biosynthesis. In parallel, ET preserved mitochondrial membrane potential, reduced mitochondrial reactive oxygen species, and enhanced the expression of key mitochondrial structural and functional markers, including COX IV, cytochrome *c*, and TOMM20. These effects are consistent with previous reports describing ET as an efficient scavenger of hydroxyl radicals, hypochlorous acid, and peroxynitrite, and support a model in which ET reinforces mitochondrial redox stability to sustain steroidogenic capacity.

Importantly, our findings complement and extend earlier studies on obesity-induced testicular dysfunction by identifying a testis-intrinsic redox-mitochondrial-steroidogenic axis that is amenable to nutritional modulation. In ex vivo human seminiferous tubule cultures, ET increased testosterone release and reduced oxidative stress, underscoring translational relevance. Given its favorable safety profile and regulatory availability as a dietary supplement, ET has potential for rapid translational testing in metabolic-disorder-associated infertility [57,67]. More broadly, our results reinforce the concept that dietary antioxidants capable of substantial tissue accumulation may confer targeted protection in organs susceptible to obesity-related oxidative damage. Integrating metabolomics with transcriptomics further enhances biomarker discovery and provides the mechanistic insight needed for precision interventions.

This study has several limitations. While we establish mechanistic links among ET, mitochondrial function, and steroidogenesis, the precise molecular targets of ET—such as direct protein interactions or thiol-dependent signaling switches—remain to be elucidated. Moreover, although ET supplementation was effective when administered concurrently with HFD, its therapeutic efficacy in established obesity warrants further investigation. Finally, the biomarker potential of testicular or circulating ET requires validation in larger, well-phenotyped human cohorts. Despite these limitations, our integrative multi-omics approach, combined with functional validation in animal and human systems, provides a coherent framework for understanding obesity-induced testicular dysfunction and identifies ET as a tractable target for translational intervention.

## 5 Conclusion

In conclusion, this study provides a systematic characterization of the testis-intrinsic redox-metabolic reprogramming triggered by chronic obesity. Our findings demonstrate that a high-fat diet (HFD) induces progressive transcriptional and metabolic remodeling, leading to the collapse of mitochondrial bioenergetics and the suppression of androgen biosynthesis within the testicular parenchyma. Mechanistically, we identified the diet-derived thiol ergothioneine (ET) as a keystone testicular-intrinsic antioxidant that undergoes progressive depletion under lipotoxic stress. ET supplementation effectively reinstates testosterone biosynthesis by reactivating the PKA-CREB-StAR signaling axis and stabilizing mitochondrial redox homeostasis. By mitigating mitochondrial superoxide accumulation and preserving membrane potential, ET relieves the critical functional bottleneck in Leydig cell steroidogenesis induced by metabolic challenge. From a scientific perspective, this work establishes ET as a novel, redox-informed biomarker of testicular health. Unlike traditional models that focus primarily on systemic HPG-axis disruption, our data highlight tissue-intrinsic metabolic failure as a proximate driver of reproductive decline in obesity. These findings expand the current understanding of the male reproductive "redox landscape" and nominate a specific metabolite whose decline heralds the onset of infertility. The translational relevance of this research is underscored by the robust protective effects of ET observed in ex vivo human seminiferous tubule cultures, where it significantly reduced oxidative stress and enhanced testosterone output. Given its exceptional safety profile and ability to accumulate in metabolically active tissues, ET represents a promising, antioxidant-guided therapeutic adjunct for the clinical management of obesity-associated male infertility.

## Ethics declarations

All procedures followed were in accordance with the ethical standards of the responsible committee on human experimentation (institutional and national) and with the Helsinki Declaration of 1964 and later versions. Informed consent to be included in the study, or the equivalent was obtained from all patients. This study was approved by the ethical committee of the Third Affiliated Hospital of Guangzhou Medical University (approval no: No. 2025025).

## CRediT authorship contribution statement

**Xiaomin Li:** Data curation, Project administration, Software, Writing – original draft, Writing – review & editing. **Jiajing Lin:** Project administration. **Man Wu:** Formal analysis, Investigation. **Feixue Han:** Methodology, Software. **Shuyan Chen:** Methodology, Software. **Hongfei Ke:** Formal analysis, Investigation. **Zhiying Huang:** Formal analysis, Investigation. **Tianwen Peng:** Data curation, Software. **Yu Lan:** Data curation, Software. **Xin Fu:** Data curation, Software. **You Che:** Investigation, Software. **Zhicong Chen:** Conceptualization, Funding acquisition, Supervision, Writing – review & editing. **Geng An:** Conceptualization, Funding acquisition, Supervision, Writing – review & editing.

## Declaration of competing interest

The authors declare that they have no known competing financial interests or personal relationships that could have appeared to influence the work reported in this paper.

## Abbreviations

**CCK-8**: Cell counting kit-8
**CASA**: Computer-assisted sperm analysis
**CAT**: Catalase
**DEGs**: Differentially expressed genes
**DHT**: Dihydrotestosterone
**DHEA**: Dehydroepiandrosterone
**DCFH-DA**: 2′,7′-dichlorofluorescin diacetate
**ET**: Ergothioneine
**EPA**: Eicosapentaenoic acid
**ECL**: Enhanced chemiluminescence
**GO**: Gene Ontology database
**GAPDH**: Glyceraldehyde-3-phosphate dehydrogenase
**HFD**: High-fat diet
**HE**: Hematoxylin and Eosin
**IC_50_**: Half-maximal inhibitory concentration
**JC-1**: 5,5′,6,6′-tetrachloro-1,1′,3,3′-tetraethylbenzimidazolyl carbocyanine iodide
**MDA**: Malondialdehyde
**ND**: Normal Diet
**OAT**: Oligoasthenoteratozoospermia
**OGTT**: oral glucose tolerance tests
**PA**: Palmitic acid
**PCA**: Principal component analysis
**SOD**: Superoxide dismutase
**TBARS**: Thiobarbituric acid reactive substances
