# Supplementary material for: Ergothioneine rescues obesity-induced testicular dysfunction via dual restoration of steroidogenesis and mitochondrial redox homeostasis
Source: Redox Biol. 2026 Feb 18;91:104090. doi: 10.1016/j.redox.2026.104090 (PMC12936830; doi:10.1016/j.redox.2026.104090)
Supplement: Multimedia component 1 [file mmc1.doc]

**Ergothioneine rescues obesity-induced testicular dysfunction via dual restoration of steroidogenesis and mitochondrial redox homeostasis**

Xiaomin Li^a†^, Jiajing Lin^a†^, Man Wu^a†^, Feixue Han^a^, Shuyan Chen^c^, Hongfei Ke^a^, Zhiying Huang^a^, Tianwen Peng^a^, Yu Lan^a^, Xin Fu^a^, You Che^b^, Zhicong Chen^a*^, Geng An^a*^

a Department of Obstetrics and Gynecology, Center for Reproductive Medicine; Guangdong Provincial Key Laboratory of Major Obstetric Diseases; Guangdong Provincial clinical Research Center for Obstetrics and Gynecology; Guangdong Hong Kong-Macao Greater Bay Area Higher Education Joint Laboratory of Maternal-Fetal Medicine; The Third Affiliated Hospital, Guangzhou Medical University, Guangzhou, China.

b HKU-Pasteur Research Pole, School of Public Health, Li Ka Shing Faculty of Medicine, The University of Hong Kong, HongKong, China.

c The First People's Hospital of Zhaoqing, Zhaoqing, Guangdong, China.

*Corresponding authors. Geng An [(angeng0505@outlook.com)](mailto:(angeng0505@outlook.com)); Zhicong Chen ([zcchen@gzhmu.edu.cn](mailto:zcchen@gzhmu.edu.cn))

^†^ These authors contributed equally to this work.

**Supplementary Figure 1**

**
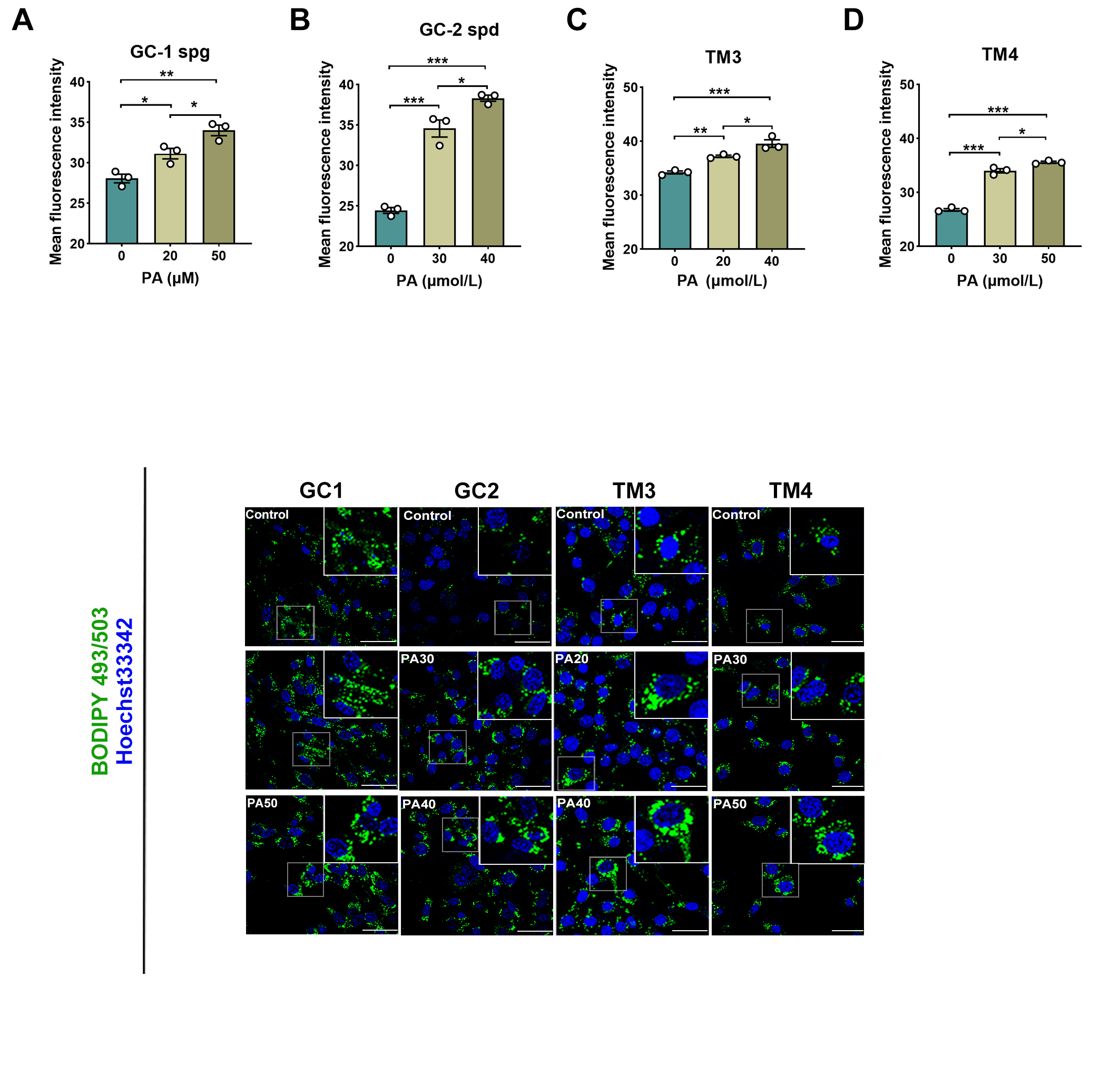
Supplementary Figure 1.** **Chronic high-fat diet progressively impairs testicular architecture and sperm quality.** (A-D) BODIPY™ 493/503 staining was used to quantitatively assess the lipid droplet content in GC-1 spg, GC-2 spd, TM3, and TM4 cells (*n*=3). Data are presented as mean ± SEM. **P* < 0.05, ***P* < 0.01, ****P* < 0.001 indicate significant differences compared to the control group.

**Table S1.** The information of the antibodies used in the present work.

| Antibody | Source | Dilutions | Catalog No. | Company |
| --- | --- | --- | --- | --- |
| GAPDH | Rabbit | 1:2000 | 10494-1-AP | Proteintech Group, Wuhan, China |
| TOMM20 | Rabbit | 1:5000 | [ab186735](https://www.abcam.cn/products/primary-antibodies/tomm20-antibody-epr15581-54-mitochondrial-marker-ab186735) | Abcam, Cambridge, UK |
| SOD2/MnSOD | Rabbit | 1:2000 | ab13533 | Abcam, Cambridge, UK |
| Cytochrome C | Rabbit | 1:3000 | ab133504 | Abcam, Cambridge, UK |
| COX IV | Rabbit | 1:1000 | ab16056 | Abcam, Cambridge, UK |
| GPX4 | Rabbit | 1:2000 | A11243 | Abclonal Technology, Wuhan, China |
| TNP1 | Rabbit | 1:500 | 17154-1-AP | Proteintech Group, Wuhan, China |
| HSD17B11 | Rabbit | 1:2000 | 16254-1-AP | Proteintech Group, Wuhan, China |
| γH2AX | Rabbit | 1:2000 | 29111-1-AP | Proteintech Group, Wuhan, China |
| P-CREB (Ser133) | Rabbit | 1:2000 | #9198 | Cell Signaling Technology, Danvers, MA, USA |
| CREB | Rabbit | 1:2000 | #9197 | Cell Signaling Technology, Danvers, MA, USA |
| P-PKA Substrate | Rabbit | 1:1000 | #9621 | Cell Signaling Technology, Danvers, MA, USA |
| StAR | Rabbit | 1:2000 | 12225-1-AP | Proteintech Group, Wuhan, China |

**Table 2. Modified Johnsen Scoring System for MTBS Evaluation**

| Score | Histological Criteria |
| --- | --- |
| 10 | Complete spermatogenesis with organized tubules and abundant spermatozoa. |
| 9 | Many spermatozoa present but with disorganized germinal epithelium. |
| 8 | Only a few spermatozoa present. |
| 7 | No spermatozoa, but many late-stage spermatids present. |
| 6 | No spermatozoa or late spermatids, but a few early spermatids present. |
| 5 | No spermatids, but many spermatocytes present. |
| 4 | Only a few spermatocytes present. |
| 3 | Only spermatogonia present. |
| 2 | No germ cells present; only Sertoli cells remain. |
| 1 | No cells present; tubular fibrosis or total atrophy. |
